# Crop classification method for multi-temporal remote sensing imagery based on a (3 + 2)D SAFPN

**DOI:** 10.3389/fpls.2026.1765836

**Published:** 2026-02-10

**Authors:** Yicong Sun, Tingting Zhao, Yue Zhang, Xia Yu, Liqian Zhang, Yunli Bai

**Affiliations:** 1 College of Computer and Information Engineering, Inner Mongolia Agricultural University, Hohhot, China; 2 Inner Mongolia Autonomous Region Key Laboratory of Big Data Research and Application of Agriculture and Animal Husbandry, Hohhot, China

**Keywords:** crop classification, deep learning, feature pyramid network, multi-temporal parcels, remote sensing

## Abstract

Accurate crop classification plays a critical role in agricultural monitoring and food security assurance. Effectively exploiting spatiotemporal information from multi-temporal remote sensing data remains a key challenge in crop mapping.This study proposes an improved neural network model, termed the (3+2)D Split-Attention Feature Pyramid Network ((3+2)D SAFPN), which is built upon a hybrid 3D–2D Feature Pyramid Network ((3+2)D FPN). The model integrates a 3D FPN to capture spatiotemporal crop dynamics, a 2D FPN to extract multi-scale spatial features, a split-attention (SA) mechanism to enhance inter-channel information interaction, and a focal loss function to improve learning performance on minority crop classes. Multi-temporal Sentinel-2 imagery acquired in 2024 was used to construct a plot-level NDVI time-series dataset for Talhu Town, Wuyuan County, Bayannur City, Inner Mongolia. The dataset was divided into training, validation, and test sets with a ratio of 6:2:2.Experimental results demonstrate that the proposed (3+2)D SAFPN model achieved overall accuracies of 89.01% and 89.06% on the test and validation sets, respectively, with Kappa coefficients of 0.82 for both sets, outperforming the original (3+2)D FPN model. Furthermore, comparative experiments conducted on the public Munich dataset indicate strong generalization ability, with accuracy improvements of 2.88% on the test set and 2.44% on the validation set compared to the baseline model.The results indicate that the (3+2)D SAFPN model effectively integrates spatial, spectral, and temporal information from multi-temporal remote sensing imagery, providing a robust and high-accuracy solution for crop classification tasks. This approach shows strong potential for large-scale agricultural monitoring applications. The source code of the proposed model is publicly available at: https://gitee.com/btgw/YicongSun/ree/(3+2)D-SAFPN_torch.

## 1 Introduction

The types and spatial distribution of crops serve as critical scientific indicators for evaluating the rational utilization of agricultural resources, providing a comprehensive reflection of crop cultivation structures (Lu et al., 2021). Timely and accurate information on crop types and their spatial distribution is essential for estimating crop yield and ensuring food security. Monitoring changes in cropping patterns over time is crucial in assisting governments and relevant agencies to formulate and adjust rational food policies, thereby safeguarding national food security (Zhang et al., 2023). Satellite remote sensing, with its ability for large-scale and long-term ground observation, enables the rapid, objective, and accurate acquisition of crop distribution data, making it a prominent research field in agricultural remote sensing (Chen et al., 2023). However, critical regions such as Talhu Town in Wuyuan County, Bayannur City in Inner Mongolia, still lack long-term crop structure maps and time-series vegetation index datasets.

There are generally two approaches to crop classification based on remote sensing imagery. The first involves aggregating spectral bands into vegetation indices that represent the physical characteristics of vegetation, among which the Normalized Difference Vegetation Index (NDVI) is the most widely adopted. The second approach utilizes multi-temporal images directly for classification (Ji et al., 2018). For instance, He et al. (2024) showed that Sentinel-2 imagery could achieve high classification accuracy by calculating vegetation indices and creating sequential input datasets, even under poor image quality conditions. Spectral, spatial, and temporal features are fundamental to extracting crop type information from remote sensing imagery (Zhang et al., 2020). Seasonality, as a key attribute of crops, makes multi-temporal remote sensing particularly effective for monitoring crop phenology and performing classification tasks (Sun et al., 2019). With the rapid advancement of remote sensing and computing technologies, scholars have extensively studied crop structures extraction using multi-source remote sensing data at various spatial resolutions, focusing on feature variables and classification algorithms (Sun et al., 2023). However, traditional shallow machine learning algorithms, such as Support Vector Machines (SVM) and Random Forests (RF), have limited nonlinear transformation layers and rely heavily on feature engineering, making it challenging to distinguish complex and heterogeneous features in images (Sheykhmousa et al., 2020). In recent years, deep learning has achieved significant breakthroughs in general computer vision and a variety of application domains. Compared with traditional machine learning techniques, deep learning demonstrates superior performance in most tasks and is gradually becoming the dominant approach in image pattern recognition (Victor et al., 2025). Convolutional Neural Networks (CNNs) are among the most successful deep learning architectures and have consistently outperformed other models in many image classification tasks (Kamilaris and Prenafeta-Boldú, 2018). For multi-temporal remote sensing imagery or time-series NDVI, 3D CNNs excel capturing features of dynamic crop growth and outperform traditional 2D CNN, SVM, and RF methods (Ferchichi et al., 2022). Researchers abroad have compared the classification performance of CNN, Recurrent Neural Networks (RNN), and hybrid networks based on multispectral time-series data, with hybrid networks demonstrating the best results (Garnot et al., 2019). Zhang et al. (2024) classified crops in the Hetao Irrigation District using time-series data and proposed a dual-path attention mechanism (DPACR) integrated with a CNN branch based on SE-ResNet, achieving an overall accuracy of 0.959. Alotaibi et al. Alotaibi et al. (2024) introduced DTODCNN-CC, a deep CNN-based method that significantly improved crop classification accuracy. Domestic studies also demonstrate the effectiveness and applicability of Geo-3D CNN and Geo-Conv1D, which incorporate spatial geographic information, in multi-temporal crop classification (Yang, 2021). Lu (2023) emphasized that in crop classification tasks using multi-source remote sensing data, deep learning models integrating depth, width, attention mechanisms, and hybrid CNN-Transformer structures show great potential for application. Although remote sensing imagery provides dynamic and temporal information, and significant progress has been made in theory, methods, and practical applications (Victor et al., 2025), 2D CNN have limitations in extracting three-dimensional features. Temporal information is often averaged and collapsed into scalars, which hinders full exploitation of this dimension (Alzubaidi et al., 2021). Although the structure of 3D CNN is well-suited to spatiotemporal representation, their high computational complexity and large parameter count make training more difficult (Guo et al., 2019). Additionally, 3D CNN may struggle to distinguish between classes with similar textures across spectral bands, which limits their widespread application in crop classification (Li et al., 2017).

The heterogeneity and fragmentation of crop landscapes in agricultural areas make it challenging to accurately capture crop features at the plot level using medium-to-low resolution imagery, thereby increasing the risk of misclassification (Zhang and Hu, 2019). To address the issues of insufficient utilization of time-series remote sensing data, the similarity of ground object features in medium-resolution imagery and the difficulty in distinguishing crop objects at the plot level, and the fact that most studies only extract a limited number of crop categories, this study constructs a plot-level time-series NDVI dataset. Combined with the (3 + 2)D SAFPN model, multi-temporal remote sensing imagery is employed for crop classification. This study focuses on exploring model optimization and improvement strategies and delving into the role of time-series information in crop classification. This work provides a new technological pathway for multi-class crop classification under conditions with limited training samples by leveraging deep learning. Not only does it offer innovative ideas and methodological references for fine-grained remote sensing classification in areas with multiple crops, it also provides scientific support and practical guidance for accurate crop censuses, land use management and the dynamic monitoring of the agricultural industry.

This paper is organised as follows: Section 2 describes the study area and the sources of the self-constructed dataset, as well as providing basic information about them. Section 3 presents the preprocessing workflow for constructing the dataset, the improved (3 + 2)D SAFPN model, and the adopted loss function. Section 4 illustrates comparative experiments between the two models on both the self-constructed and public datasets, as well as ablation studies. It also maps crop distributions and estimates the cultivated area in the study region based on the results of the classification. Sections 5 and 6 discuss the research findings and conclude the paper, respectively.

### 2 Study area and data

### 2.1 Overview of the study area

**Figure 1:**
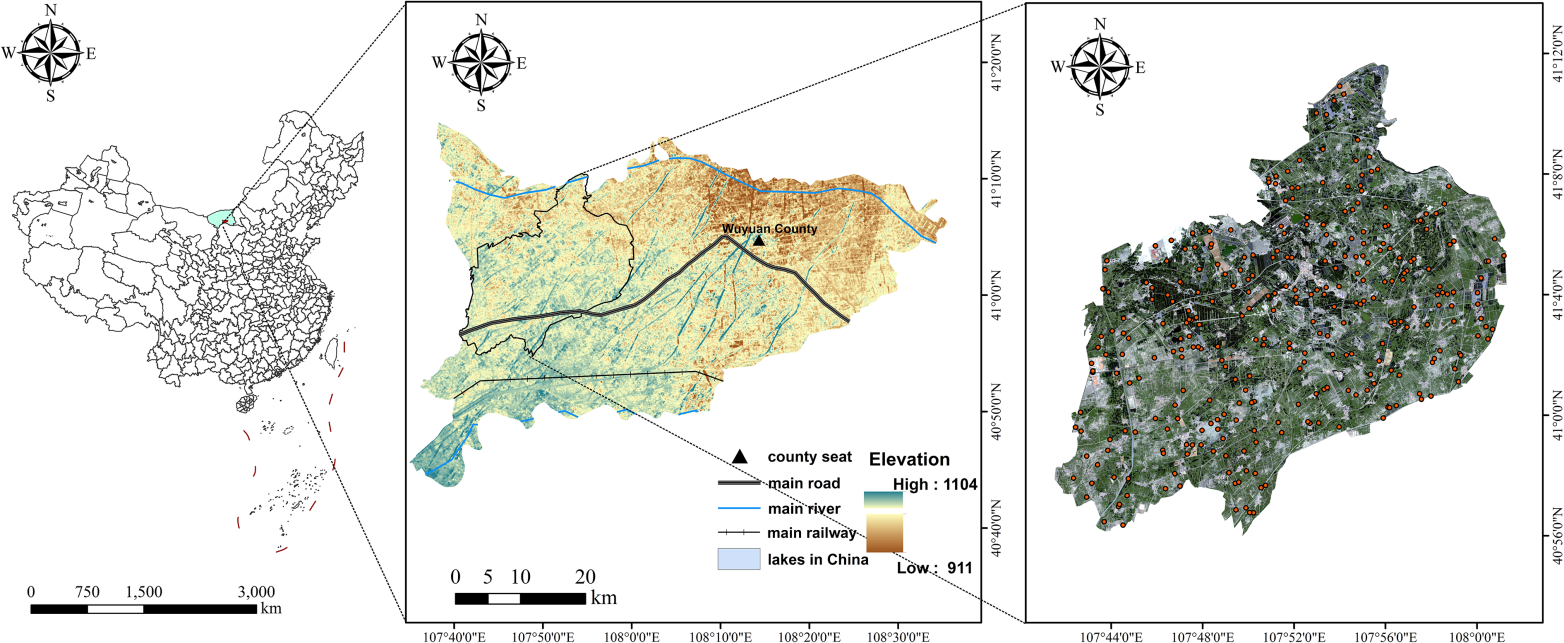
Overview of Talhu Town and the spatial distribution of sample sites.

Talhu Town (**Figure 1**) is located in Wuyuan County, Bayannur City, Inner Mongolia Autonomous Region, China. Its geographic coordinates range from 107°41’E to 108°01’E and 40°56’N to 41°11’N. It covers a total area of approximately 428.47 km² (National Bureau of Statistics of China, 2020). Situated on the northern bank of the middle reaches of the Yellow River, the study area lies within the Hetao Plain. This region is characterized primarily by flat terrain, accounting for 91.8% of the total land area. The region’s fertile soil makes it highly suitable for agricultural and pastoral activities. Talhu Town experiences a temperate continental monsoon climate, characterised by significant temperature variation, abundant sunshine, high evaporation rates, and low but concentrated precipitation (Ministry of Civil Affairs of the People’s Republic of China, 2018). The pronounced diurnal temperature range and climatic conditions are favourable for the growth of various crops. Major staple crops include maize, wheat, and potatoes, while economic crops consist mainly of sunflower, tomato, zucchini, and sugar beet. The study area exhibits unique climatic and geomorphological characteristics, including large, contiguous, well-leveled farmland. The cultivated land is systematically managed, demonstrating a high degree of crop diversification and mechanized farming. These features make Talhu Town an ideal and representative area for remote sensing monitoring and precision agriculture applications, particularly for evaluating crop classification algorithms.

Based on agricultural statistics from the Wuyuan County Government (http://www.wuyuan.gov.cn/bsfw/) and the spatial distribution of major crops in the study area, this research focuses on classifying seven crop types: maize, sunflower, wheat, honeydew melon, tomato, zucchini, and sugar beet. Other non-agricultural land cover types, such as urban areas, greenhouses, sandy land, and water bodies, are collectively categorized as “others” (**Table 1**).

**Table 1:** Land cover types and number of delineated plots.

Index	Crops and other ground objects	Number of training parcels	Number of testing parcels	Verify the number of parcels
1	Maize	4691	1563	1565
2	Sunflower			
3	Wheat			
4	Honeydew melon			
5	Tomato			
6	Zucchini			
7	Sugar beet			
0	Others			

### 2.2 Remote sensing imagery data

The remote sensing data used in this study were acquired from the Sentinel-2 satellite system, which is part of the Copernicus Earth Observation Programme run by the European Space Agency (ESA). This system consists of two satellites, Sentinel-2A and Sentinel-2B, which are equipped with multispectral imaging capabilities. Due to its high spatial resolution and wide spectral coverage, Sentinel-2 imagery is widely used in applications such as agricultural monitoring, land cover classification and natural disaster assessment. It provides essential support for the dynamic observation of Earth’s resources and environment.

To ensure that the time-series data adequately capture crop growth dynamics, the construction of the full-year 2024 time-series dataset for Talhu Town comprehensively considered multiple factors, including the spatial coverage of the study area, the phenological stages of major crops, image spatial projection (WGS_1984_UTM_Zone_48N), acquisition dates, and cloud coverage (all below 10%). Ultimately, 20 optimal acquisition dates, comprising a total of 40 Sentinel-2 scenes that fully cover Talhu Town throughout the year, were selected as the study dataset. All Sentinel-2 images were obtained from the European Space Agency’s Copernicus Open Access Hub (https://dataspace.copernicus.eu/).

The temporal distribution of the selected 20 image acquisitions closely corresponds to the key phenological stages of the major crops in the study area, such as emergence, vigorous vegetative growth, and the pre-harvest stage. These time points effectively capture vegetation condition variations across different growth phases, thereby providing sufficient representational capacity for time-series feature modeling. In addition, the selected images ensure low cloud contamination and complete spatial coverage, which further enhances the reliability of the time-series dataset.

### 2.3 Publicly available dataset

This study conducts a comparative validation experiment using the publicly available Munich dataset and a self-constructed time-series NDVI dataset constructed for Talhu Town in Wuyuan County, Bayannur City. The Munich dataset comprises 48×48 pixel image patches containing 13 Sentinel-2 spectral bands and covers an area of approximately 102 km × 42 km in northern Munich, Germany. In our experiments, the split0 subset of the Munich dataset was used, which includes 17 crop categories and contains 6534, 1944, and 2016 plots for training, validation, and testing, respectively. The Munich dataset was chosen as a benchmark due to its comprehensive combination of remote sensing imagery and ground truth survey data. Moreover, as a widely recognized public dataset, it provides an objective basis for evaluating model performance and enables a direct comparison of classification accuracy with the Talhu Town dataset. Detailed information on the Munich dataset can be found in Rußwurm (2025).

### 2.4 Sample dataset

**Figure 2:**
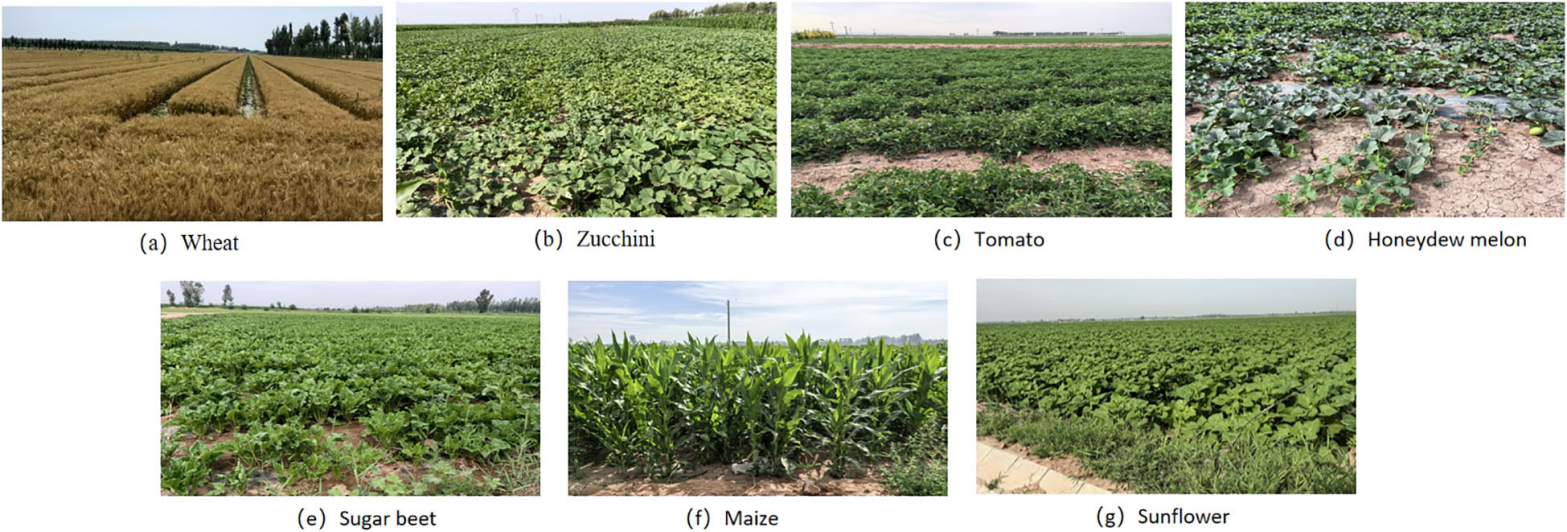
Example images of crop growth conditions. Figures **(a–g)** show the in-field photographs of crops collected during field sampling: Wheat, Zucchini, Tomato, Honeydew Melon, Sugar Beet, Maize, and Sunflower, respectively.

Field sampling in Talhu Town was conducted using centimeter-level Real-Time Kinematic (RTK) positioning technology (provided by the Qianxun RTK receiver) in conjunction with the Ovi interactive mapping software. Sampling points were distributed along the diagonals of crop plots, with a particular focus on densely cultivated areas to ensure sample representativeness. High-precision GPS equipment was used to record geographic coordinates. The selection of sampling points adhered to the following principles: consistent crop type, uniform crop growth status, coverage of all major crop types in the study area, and photo documentation of each sample for subsequent verification (see **Figure 2**). Key attributes recorded at each sample point included crop type, sample ID, growth status, and representative non-agricultural land types (urban areas, greenhouses, water bodies, and sandy land), along with detailed latitude and longitude information. Field data collection took place from 7 to 13 July 2024, during which 200 valid samples were evenly distributed across the entire study area (see **Figure 1**).

## 3 Methodology

**Figure 3:**
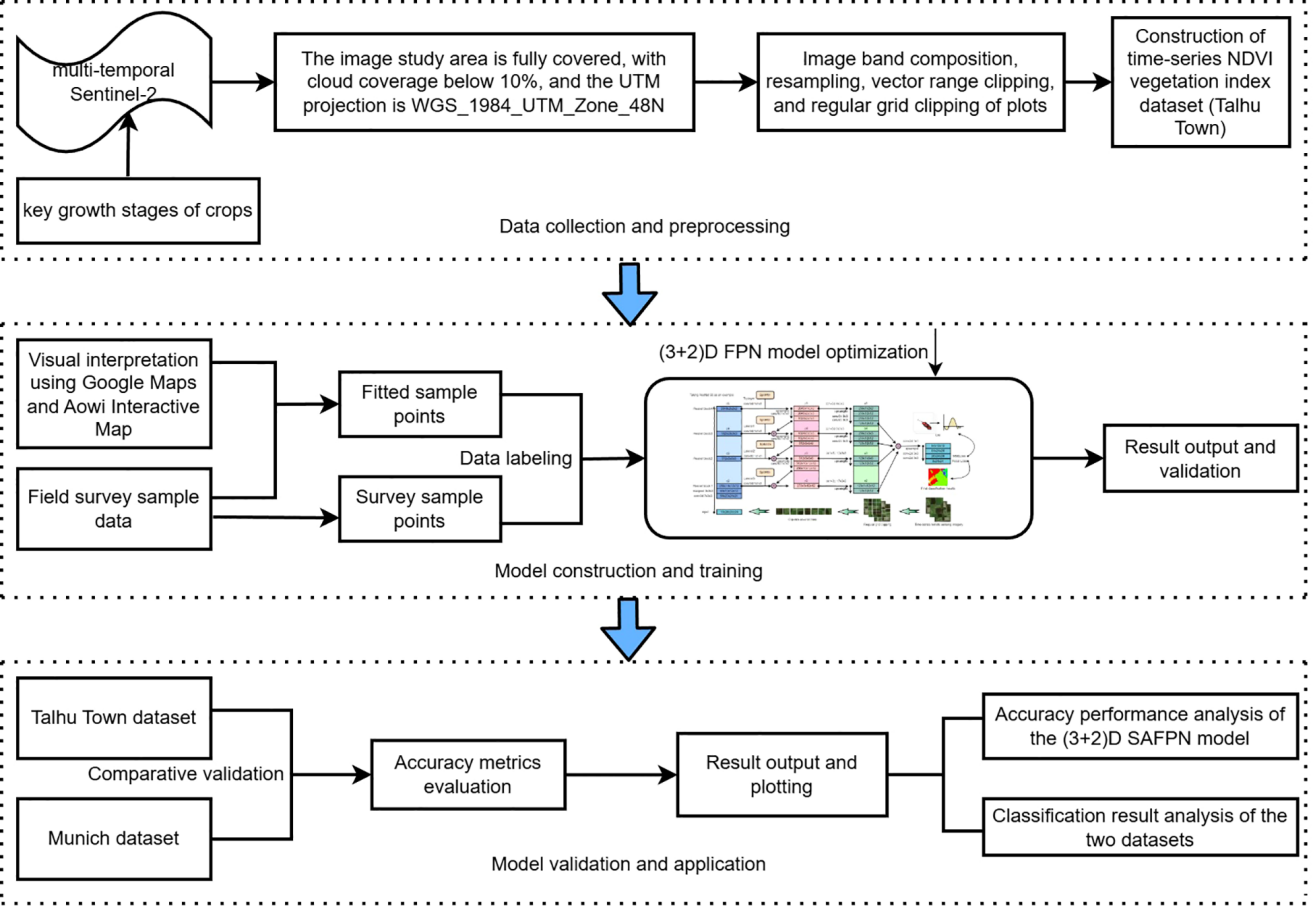
Technical flowchart.

This study utilized multi-temporal Sentinel-2 imagery to construct a time-series NDVI crop classification dataset for Talhu Town. Preprocessing steps included band composition, vector clipping, and regular grid partitioning. NDVI features were extracted based on vegetation phenology and labeled using crop categories sampled in the field. Each pixel was labeled according to the dominant crop type throughout the year rather than at a single temporal snapshot. To address the limited number of field samples, SVM classification was employed to propagate labels from the ground-truth samples to the entire study region, assigning a crop category label to each pixel. Inspired by the flexibility of SA) mechanisms in feature extraction, the SA block was embedded into a (3 + 2)D FPN architecture to enhance multi-scale and multi-channel feature fusion capabilities while maintaining computational efficiency. Additionally, to mitigate class imbalance, the model incorporated the Focal Loss function in the classification output layer to improve recognition performance for hard and underrepresented categories. The model’s performance was evaluated using both the public Munich dataset and the Talhu Town dataset to verify its effectiveness in identifying crop types using time-series remote sensing data. The complete technical framework is illustrated in **Figure 3**.

### 3.1 Construction of the time-series NDVI dataset for Talhu Town

To prevent potential spatial and temporal information leakage and to ensure the objectivity and reliability of model evaluation, the training, validation, and test sets were strictly separated during the dataset construction and splitting stages. The construction of a new region-specific dataset not only enriches the diversity of training samples but also facilitates a systematic evaluation of the model’s generalization capability across different spatial scenarios.

Specifically, the remote sensing imagery of Talhu Town was partitioned into 7819 spatially independent patch units using a regular grid of 24 × 24 pixels. Each patch corresponds to a fixed spatial location and contains 20 high-quality Sentinel-2 multispectral image acquisitions from 2024, thereby forming a complete time-series sample. Each acquisition includes four 10 m resolution bands (B2, B3, B4, and B8), six 20 m resolution bands (B5, B6, B7, B8A, B11, and B12), and three 60 m resolution bands (B1, B9, and B10), along with the corresponding crop type label map.

During dataset splitting, patch units were used as the minimum splitting unit to ensure that the time-series images from the same spatial patch do not appear in multiple data subsets, thereby effectively avoiding both spatial and temporal information leakage. All patch samples were randomly divided into 4691 training samples, 1563 validation samples, and 1565 test samples according to a ratio of 6:2:2 (**Table 1**). The training set was used exclusively for model parameter learning, the validation set for model tuning and early stopping, and the test set remained completely independent for final performance evaluation.

The NDVI is widely used to assess vegetation health by comparing the difference between near-infrared reflectance, which vegetation strongly reflects, and red reflectance, which vegetation strongly absorbs (Xu et al., 2019). The NDVI is calculated using Equation 1:

(1) $$NDVI=\frac{NIR-RED}{NIR+RED}=\frac{B8-B4}{B8+B4}$$

In the formulas, NIR refers to the reflectance in the near-infrared band, which is highly sensitive to vegetation, while RED refers to the reflectance in the red band, which is strongly absorbed by chlorophyll in plant leaves. The NIR and Red bands both correspond to specific spectral bands provided by the Sentinel-2 satellite.

In this study, the NDVI was computed on a per-pixel basis from the full-year 2024 Sentinel-2 imagery to construct a time-series NDVI dataset for the Talhu Town region. NDVI was selected for the following reasons: (1) High sensitivity to vegetation conditions: NDVI effectively reflects vegetation density and growth vigor, providing stable discrimination across different crop types and growth stages. (2) Data availability and consistency: Sentinel-2 offers near-infrared and red bands with high spatial resolution (10 m) and high temporal resolution (5 days), enabling straightforward NDVI computation with reliable and consistent results. (3) Extensive application foundation: NDVI is a standard index in remote sensing–based vegetation monitoring and crop classification, ensuring comparability with existing studies and supporting long-term time-series analysis. (4) Suitability for time-series analysis: When constructing a full-year time-series dataset, NDVI continuously captures the dynamic evolution of crops from sowing and growth to harvesting, thereby providing stable spatiotemporal features for model learning.

The resulting dataset not only represents the dynamic crop growth process but also assigns a dominant crop type label to each pixel for the entire year, without including explicit sowing or harvesting date information. Based on the NDVI time-series features, spatial and temporal patterns of crop growth stages can be efficiently captured, providing reliable inputs for subsequent training of the proposed (3 + 2)D SAFPN model.

### 3.2 Convolutional neural network

#### 3.2.1 (3 + 2)D FPN

The (3 + 2)D FPN is a variant of the conventional FPN designed to jointly leverage both 3D and 2D feature extraction. This makes it particularly suitable for tasks involving spatiotemporal data, such as video analysis and time-series remote sensing (Gallo et al., 2021). Originally proposed in 2017, the FPN architecture addresses the limitations of traditional CNNs in detecting objects at multiple scales (Lin et al., 2017). Standard CNNs typically perform object detection on the final feature map, which often fails to handle large and small objects effectively. FPN enhances detection performance by aggregating features across multiple layers to capture information at various scales. 3D convolutions are adept at processing spatiotemporal data, enabling simultaneous extraction of spatial and temporal features by treating the input as a volumetric sequence (Choy et al., 2019). 2D convolutions, by contrast, focus purely on spatial features and are computationally more efficient, making them well-suited for tasks with detailed spatial information such as image segmentation and object detection (Gu et al., 2022). The (3 + 2)D FPN combines the strengths of both approaches by first applying 3D convolutions to extract temporal-spatial features and subsequently integrating 2D convolution layers to refine spatial representations. The resulting multi-scale feature hierarchy captures rich semantic features across different levels, with 3D features typically being transformed into 2D feature maps before fusion.

#### 3.2.2 (3 + 2)D SAFPN

This study proposes a novel architecture based on the (3 + 2)D FPN and incorporates a SA mechanism to enhance the network’s feature selection capability. The SA module enables the model to dynamically adjust attention weights across different channels, thereby improving the extraction of key information across multiple spatial scales and temporal dimensions. Originating from the ResNeSt network, the SA module splits the input features into multiple groups, applies attention independently to each group, and then fuses the reweighted group features, effectively enhancing the overall representational capacity (Zhang et al., 2022).

In the (3 + 2)D SAFPN, the input images are first processed through a series of convolutional layers with progressive downsampling to generate feature maps at different resolutions and semantic depths (c2, c3, c4, c5). Specifically, each convolutional block consists of two 3 × 3 convolutional layers, with output channels sequentially set to 256, 512, 1024, and 2048. Downsampling is achieved with a stride of 2, and each convolutional layer is followed by ReLU activation and batch normalization. The resulting multi-scale feature maps are then fed into the FPN for top-down feature fusion.

At each scale, the SA module divides the channels into four groups (group = 4). Global average pooling is applied to each group to generate a global feature descriptor, which is then passed through two fully connected layers (with the intermediate hidden layer dimension set to twice the number of channels per group) to generate attention weights. These weights are normalized via a Softmax function and applied back to the corresponding group features, enhancing important channels while suppressing less informative ones. This mechanism allows the network to adaptively select key features during multi-scale feature fusion, improving the representational power of features at different layers. This is particularly beneficial for crop classification tasks where spectral similarity and complex phenological variations exist.

Within the FPN module, feature maps from different scales are progressively upsampled and combined through lateral connections. During this process, the SA mechanism adaptively adjusts the features at each layer, resulting in fused feature maps with higher selectivity and discriminative ability.

**Figure 4:**
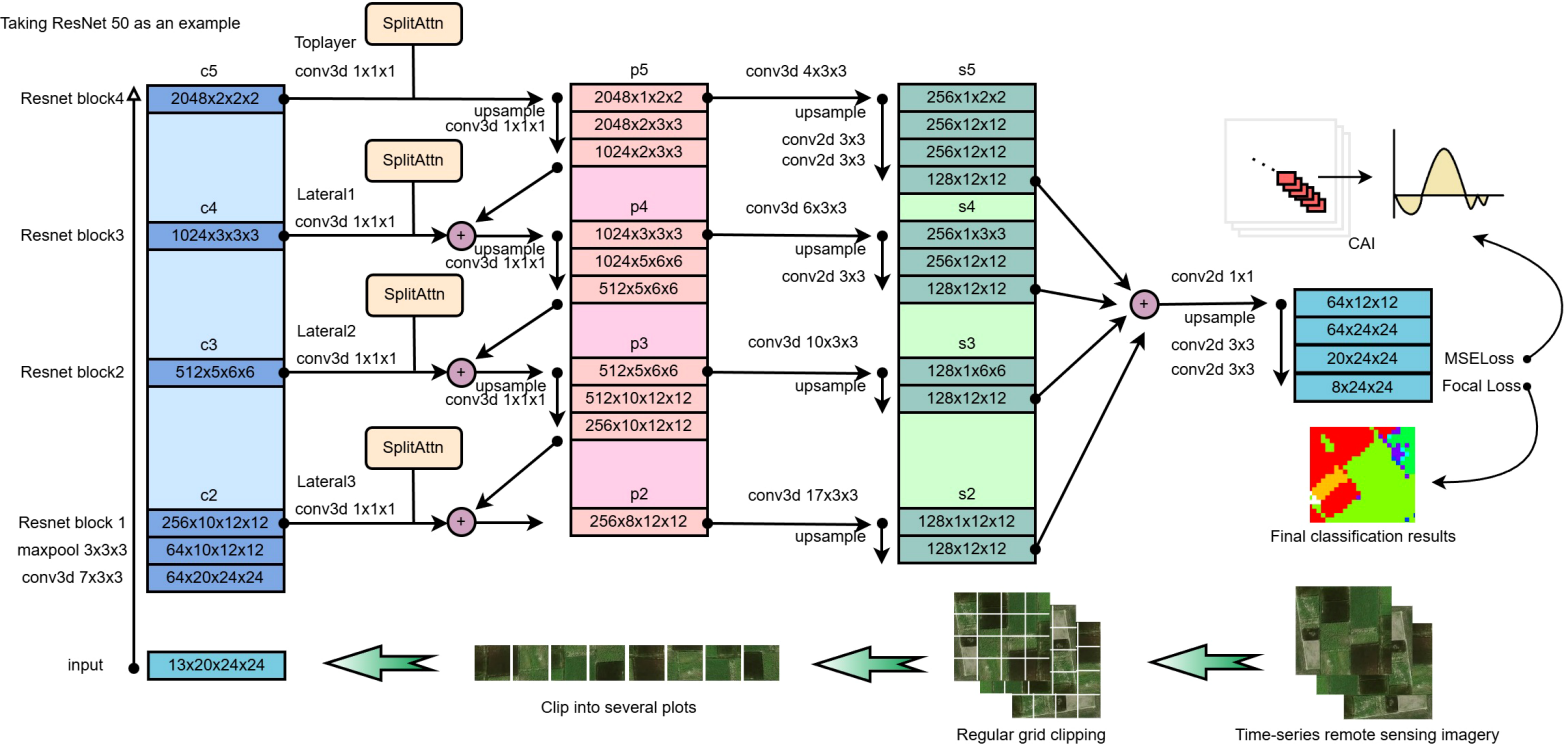
(3 + 2)D SAFPN model architecture diagram.

The overall architecture is illustrated in **Figure 4**, comprising 3D convolutions to capture temporal and spatial information, 2D convolutions for spatial-scale feature extraction, and SA modules for enhanced feature selection. Through this design, the (3 + 2)D SAFPN effectively integrates spatiotemporal information and multi-scale features, enabling accurate classification of crops from complex multi-temporal remote sensing data.

**Figure 5:**
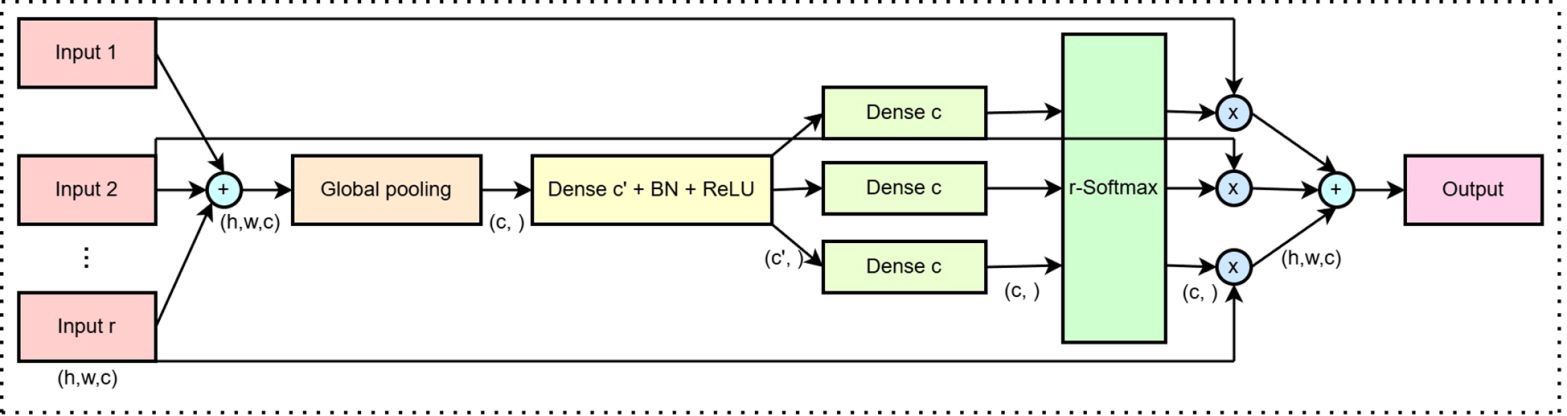
Split-Attention module architecture diagram.

The structure of the Split-Attention module is shown in **Figure 5**. This architecture operates within a cardinal group, which typically refers to a set of features or channels that are processed together in a neural network. Suppose the input feature map is denoted as $X\in {\text{R}}^{H\times W\times C}$, where H is the height, W is the width, and C is the number of channels. The input is divided into r branches $\left(Input_{1},\;Input_{2},\ldots ,Input_{r}\right)$, with each branch having a shape of (h,w,c), where c= represents the number of channels per branch and K denotes the number of groups. The features from all branches are summed to obtain the aggregated feature $X_{\text{agg}}$, as shown in Equation 2. Where $X_{i}\in {\text{R}}^{H\times W\times c}$ denotes the feature representation of each branch.

(2) $$X_{\text{agg}}=\sum_{i=1}^{r}X_{i}$$

Through global pooling, $X_{\text{agg}}$ is reduced from a 3D feature map of H×W×c to 1×1× c, representing the global descriptor of each channel. The global pooling operation is defined in Equation 3.

(3) $$X_{\text{pool}}=\text{GlobalPooling}(X_{\text{agg}})\in {\text{R}}^{c}$$

A dense layer is used to project $X_{\text{pool}}$ into a lower-dimensional space $c^{\prime }$, followed by batch normalization (BN) and a ReLU activation function, as described in Equation 4.

(4) $$Y=\text{ReLU}(\text{BN}(\text{Dense}(X_{\text{pool}})))$$

The feature Y is projected back to its original dimension c through multiple parallel dense layers, and attention weights are assigned using r-Softmax. The r-Softmax operation normalizes the weights across all branches using the Softmax function, ensuring that the sum of weights equals 1, as shown in Equation 5. Here, $A_{i}\in {\text{R}}^{c}$ denotes the attention weight for the i-th branch.

(5) $$A_{i}=\text{Softmax}(\text{Dense}(Y))$$

Each input branch $X_{i}$ is multiplied by its corresponding attention weight $A_{i}$, and all the weighted feature branches are summed to obtain the final output feature $X_{\text{output}}$, as shown in Equation 6, where “ ·“ denotes element-wise multiplication.

(6) $$X_{\text{output}}=\sum_{i=1}^{r}A_{i}\cdot X_{i}$$

The shape of the final output feature $X_{\text{output}}$ is (h,w,c), which is consistent with that of each input branch $X_{i}$. Through the SA module, the weights of each feature subgroup are dynamically adjusted, thereby enhancing the network’s ability to represent informative features.

The SA module dynamically reweights sub-channel features, enabling the network to automatically focus on informative channels while suppressing redundant or noisy features. This capability is particularly important for multi-temporal remote sensing data, where crop features change over time but may exhibit high spectral similarity. In the (3 + 2)D SAFPN, the input features encompass both the temporal dimension (processed via 3D convolutions) and the spatial dimension (processed via 2D convolutions). By applying adaptive weighting to the sub-channels, the SA module can capture salient information across time steps and spatial scales, thereby enhancing the representation of spatiotemporal features.

Visualization of the attention weights $A_{i}$ for each sub-branch reveals that the network assigns higher weights during critical crop growth stages, such as emergence, vigorous vegetative growth, and the pre-harvest period, while lower weights are assigned during periods with minimal spectral or phenological differences. This observation not only validates the effectiveness of the SA module on spatiotemporal data but also provides interpretability for the model’s decision-making process.

In summary, through dynamic channel weighting and multi-branch aggregation, the SA module enables the network to automatically extract key information from spatiotemporal data, thereby improving both the accuracy and robustness of multi-temporal remote sensing crop classification.

### 3.3 Loss function

This study uses Mean Squared Error (MSE) Loss for the regression task and Focal Loss for the classification tasks in the model. In the final layer of the model, the NDVI prediction layer, MSE Loss is used to learn the NDVI values of each pixel in the input time series. The Class Activation Interval (CAI) is used to determine the time periods in the input time series that are most decisive for predicting the output class. Through MSE loss learning in the NDVI layer, the model can accurately predict the NDVI variations over time and identify key growth stages of specific classes, such as certain crops. The MSE loss Equation 7 is as follows:

(7) $$L_{\text{MSE}}=\frac{1}{n}\sum_{i,j}{\left(y_{i,j}-t_{i,j}\right)}^{2}$$

Where $y_{i,j}$ is the predicted NDVI value for the i, j-th pixel, $t_{i,j}$ is the true NDVI value for pixel i, j, and n is the total number of pixels in the image. By computing the squared differences between the predicted and true values, MSE loss reflects the degree of fit between the model’s predictions and the actual NDVI values. A smaller loss indicates that the predicted NDVI is closer to the true value.

Focal Loss plays a crucial role in the semantic segmentation classification task by helping the model to focus more on the learning of rare classes. In crop classification tasks, Focal Loss significantly improves classification performance balancing across different classes. Focal Loss improves and optimises Cross-Entropy (CE) Loss, and the Equation 8 is as follows:

(8) $$FocalLoss(p_{t})=-\alpha_{t}{\left(1-p_{t}\right)}^{\gamma }log(p_{t})$$

where $p_{t}$ is the predicted probability for the true class, as defined in Equation 9:

(9) $$p_{t}=\{\begin{array}{c} p\;\;\;\;\;\;\text{if label is positive class}\\ 1-p\;\;\;\text{if label is negative class} \end{array}$$

The value of p ranges between [0,1], representing the weight ratio of positive and negative samples. $\alpha_{t}$ is a balancing factor that helps adjust the influence of positive and negative samples. γ is the focusing parameter, controlling the loss decay rate for difficult-to-classify samples, typically set to 2. The factor ${\left(1-pt\right)}^{\gamma }$ serves as an adjustment factor to amplify the loss weight for hard-to-classify samples.

The collaborative use of MSE Loss and Focal Loss allows the model to both capture the NDVI variation trends in the time series and classify each pixel in the sample accurately.

### 3.4 Evaluation metrics for classification accuracy

In order to evaluate the performance of deep learning algorithms in multi-crop classification tasks within the Talhu Town study area, 20% of the parcel samples were selected from the test and validation sets to construct confusion matrices (Powers, 2020). Based on these matrices, a series of accuracy evaluation metrics were calculated, including Overall Accuracy (OA), Precision (P), Recall (R), F1-score, and the Kappa coefficient (McHugh, 2012). These metrics effectively reflect both the overall and class-specific performance of the model in multi-class classification scenarios. The detailed formulations are presented in (Equations 10–15).

(10) $$\text{OA}=\frac{\sum_{i=1}^{n}a_{ii}}{N}$$

(11) $$P_{i}=\frac{a_{ii}}{a_{+i}}$$

(12) $$R_{i}=\frac{a_{ii}}{a_{+i}}$$

(13) $$\text{F}1-{\text{score}}_{i}=\frac{2\cdot {\text{P}}_{i}\cdot {\text{R}}_{i}}{{\text{P}}_{i}+{\text{R}}_{i}}$$

(14) $$\text{Kappa}=\frac{\text{OA}-p_{c}}{1-p_{c}}$$

(15) $$p_{c}=\frac{\sum_{i=1}^{n}(a_{i+}\cdot a_{+i})}{N^{2}}$$

In these equations, $a_{ii}$ denotes the diagonal elements of the confusion matrix; $a_{i+}$ refers to the total number of ground truth pixels for class i; $a_{+i}$ indicates the total number of pixels predicted as class i; and N is the total number of pixels (Fang et al., 2024).

## 4 Results and analysis

### 4.1 Environment configuration and model training

The experiments were conducted using the Python 3.8 programming language and the TensorFlow 2.5 deep learning framework. The computational environment was equipped with an Intel Xeon Gold 6252 processor, an NVIDIA Tesla V100S GPU, and 32 GB of RAM.

Experiments were performed on both the Munich and Talhu Town datasets. During training, an NDVI loss was introduced to help the model learn to recognize temporal NDVI variation patterns. The number of training epochs was set to 300, with a batch size of 2. ResNet50 and ResNet101 were employed as the backbone networks. The Stochastic Gradient Descent (SGD) optimizer with a momentum of 0.9 was adopted (Ye et al., 2023), with a weight decay coefficient of 0.001 and an initial learning rate of 0.01. The temporal lengths of the Munich and Talhu Town datasets consisted of 30 and 20 time steps, respectively. After training the modules, pretrained weights were loaded, and CAIs were extracted from the NDVI loss layer to analyze the dynamic growth characteristics and abnormal variations of different crops.

### 4.2 Comparative experiments

#### 4.2.1 Comparative performance of different model architectures on the munich dataset

To evaluate the effectiveness of the proposed model in multi-temporal remote sensing crop classification, we conducted a comparative study on the Munich crop dataset, assessing the classification performance of the proposed (3 + 2)D SAFPN against the baseline (3 + 2)D FPN under different backbone network depths. The results are summarized in **Table 2**. The (3 + 2)D SAFPN consistently outperformed the baseline model on both the validation and test sets.

**Table 2:** Comparative performance of different model architectures on the munich dataset.

Dataset	Model	Model_depth	Number of crop categories	OA of the test set (%)	OA of the val set (%)	GPU MEM(MiB)
Munich	(3 + 2)D FPN	50	17	90.15	91.38	2758
		101		92.94	93.55	3468
	(3 + 2)D SAFPN	50		95.82	95.99	2950
		101		95.79	95.97	3824

When using ResNet-50 as the backbone, the (3 + 2)D FPN achieved an overall accuracy (OA) of 90.15% on the test set, whereas the (3 + 2)D SAFPN improved OA to 95.82%, representing an absolute gain of 5.67 percentage points. With the backbone depth increased to ResNet-101, the (3 + 2)D SAFPN maintained high classification performance, achieving a test set OA of 95.79%, which corresponds to a 2.85 percentage point improvement over the FPN model of the same depth. These results indicate that the spatially adaptive feature fusion mechanism effectively enhances the discriminative capability of multi-temporal features, thereby significantly improving crop classification accuracy.

Furthermore, as the backbone depth increased from ResNet-50 to ResNet-101, the classification performance of the baseline (3 + 2)D FPN improved noticeably, whereas the (3 + 2)D SAFPN exhibited relatively stable performance across different network depths. This suggests that the proposed (3 + 2)D SAFPN is able to fully exploit multi-scale spatiotemporal feature information even with a shallower backbone, demonstrating lower sensitivity to network depth and higher structural efficiency.

In terms of computational cost, the (3 + 2)D SAFPN introduced only a modest increase in GPU memory usage compared with the baseline (3 + 2)D FPN, while achieving a substantial gain in accuracy. Considering both classification performance and computational efficiency, the proposed (3 + 2)D SAFPN exhibits an excellent accuracy–efficiency trade-off for multi-temporal crop classification, highlighting its potential for large-scale agricultural remote sensing crop mapping applications.

#### 4.2.2 Comparative performance of different model architectures on the Talhu town dataset

To further evaluate the generalization capability of the proposed model across different crop types and regional scenarios, comparative experiments were conducted on the self-constructed Talhu Town crop dataset, comparing the (3 + 2)D SAFPN with the baseline (3 + 2)D FPN. The dataset contains seven crop classes, and the results are summarized in **Table 3**.

**Table 3:** Comparative performance of different model architectures on the talhu town dataset.

Dataset	Model	Model_depth	Number of crop categories	OA of the test set (%)	OA of the val set (%)	GPU MEM(MiB)
Talhu Town	(3 + 2)D FPN	50	7	84.72	84.88	2411
		101		86.65	86.66	3064
	(3 + 2)D SAFPN	50		89.01	89.06	2472
		101		89.00	89.04	3151

The experimental results indicate that, under different backbone network depths, the proposed (3 + 2)D SAFPN consistently outperformed the baseline (3 + 2)D FPN on both the validation and test sets. When using ResNet-50 as the backbone, the (3 + 2)D FPN achieved an overall accuracy (OA) of 84.72% on the test set, whereas the (3 + 2)D SAFPN improved OA to 89.01%, corresponding to an absolute gain of 4.29 percentage points. With the backbone depth increased to ResNet-101, the (3 + 2)D SAFPN achieved a test set OA of 89.00%, which represents a 2.35 percentage point improvement over the corresponding (3 + 2)D FPN.

Consistent with the results on the Munich dataset, the baseline (3 + 2)D FPN exhibited moderate performance gains with increased backbone depth, while the (3 + 2)D SAFPN demonstrated relatively stable classification performance across different network depths, indicating lower sensitivity to backbone depth. This suggests that the spatially adaptive feature fusion mechanism can effectively model the spatiotemporal discriminative features of multi-temporal crops even with shallower networks, reducing reliance on deep semantic features.

In terms of computational resources, the (3 + 2)D SAFPN introduced only a modest increase in GPU memory usage compared with the baseline (3 + 2)D FPN, with an additional ~61 MiB for ResNet-50 and ~87 MiB for ResNet-101. Considering both accuracy improvement and computational cost, the proposed (3 + 2)D SAFPN demonstrates a favorable accuracy–efficiency trade-off on the Talhu Town dataset, further validating its applicability and stability across different regions and crop type scales.

#### 4.2.3 Ablation study

To further evaluate the effectiveness of the SA module and Focal Loss in multi-temporal crop classification, an ablation study was conducted on the Talhu Town dataset. All experiments used ResNet-50 as the backbone, and the classification performance of different model architectures and loss function combinations was systematically compared, with results summarized in **Table 4**.

**Table 4:** Ablation study.

Model	Focal loss	OA of the test set (%)	OA of the val set (%)
(3 + 2)D FPN	None	84.72	84.88
(3 + 2)D FPN +SA	None	86.61	86.73
(3 + 2)D FPN +Focal loss	γ=2, α=[0.05,0.1,0.3,0.3,0.3,0.1,0.1,0.1]	87.22	87.56
(3 + 2)D FPN +SA+Focal loss ((3 + 2)D SAFPN)	γ=2, α=[0.05,0.1,0.3,0.3,0.3,0.1,0.1,0.1]	89.01	89.06

The results show that the baseline (3 + 2)D FPN achieved an overall accuracy (OA) of 84.72% on the test set. By introducing only the SA module into the FPN structure, without addressing class imbalance, the test set OA increased to 86.61%, and the validation set OA increased to 86.73%. This indicates that the SA module can effectively enhance the spatial representation of multi-scale temporal features, positively contributing to crop class discrimination.

Further incorporating Focal Loss to mitigate class imbalance led to an improvement in test set OA to 87.22% for the (3 + 2)D FPN. This result demonstrates that Focal Loss assigns higher weights to hard-to-classify samples and minority classes, partially alleviating the model’s recognition bias towards uneven crop distributions—a scenario commonly encountered in agricultural remote sensing, where planting areas of different crops vary widely.

When both the SA module and Focal Loss were integrated, forming the complete (3 + 2)D SAFPN, the model achieved the best classification performance on the Talhu Town dataset, with test and validation set OA reaching 89.01% and 89.06%, respectively. Compared with the baseline (3 + 2)D FPN, the test set OA increased by 4.29 percentage points, significantly higher than the gains obtained by introducing either the SA module or Focal Loss alone.

Overall, the ablation study indicates that the SA module and Focal Loss play complementary roles in multi-temporal crop classification: the former enhances spatiotemporal feature representation, while the latter effectively mitigates class imbalance. Their synergistic effect substantially improves the model’s overall discriminative capability, validating the rationale and effectiveness of the proposed (3 + 2)D SAFPN architecture.

### 4.3 Evaluation of classification results from the model

**Table 5** presents the classification evaluation results for the two models on the Munich dataset. The (3 + 2)D SAFPN model with a ResNet50 backbone achieved higher overall accuracies of 95.99% and 95.82% on the validation and test sets, respectively, surpassing the baseline model’s results of 93.55% and 92.94%. The improvement in the Kappa coefficient further confirms the effectiveness of the proposed model. For major crop types such as rapeseed, hops, and winter wheat, both models attained high F1-scores exceeding 0.94. Notably, the (3 + 2)D SAFPN model showed significant improvements in R and F1-score for rare classes such as winter rye and winter spelt, with F1-scores on the validation set increasing from 0.45–0.58 to 0.71–0.85. However, the classification accuracy for these rare categories remained lower than that of the dominant crops. This may be attributed to the similar spectral and temporal responses of these crops under certain growth conditions, possibly due to differences in soil moisture and nutrient availability across field types (Liu et al., 2024), which increases classification difficulty. The improvements in R and F1-score achieved by the (3 + 2)D SAFPN model can be attributed to the enhanced feature representation and class imbalance handling capabilities provided by the SA mechanism and Focal Loss.

**Table 5:** Performance comparison of the (3 + 2)D FPN and (3 + 2)D SAFPN models on the test and validation sets of the Munich dataset.

Model	(3 + 2) D FPN						(3 + 2) D SAFPN					
Model_depth	101						50					
Dataset	Munich (val set)			Munich (test set)			Munich (val set)			Munich (test set)		
Accuracy metric	P	R	F1	P	R	F1	P	R	F1	P	R	F1
Sugar beet	0.91	0.94	0.92	0.96	0.90	0.93	0.97	0.95	0.96	0.96	0.97	0.96
Summer oat	0.79	0.68	0.73	0.83	0.70	0.76	0.87	0.78	0.82	0.86	0.81	0.83
Meadow	0.90	0.87	0.88	0.93	0.90	0.91	0.96	0.93	0.94	0.93	0.92	0.93
Rapeseed	0.96	0.98	0.97	0.96	0.98	0.97	0.97	0.98	0.98	0.97	0.98	0.98
Hop	0.95	0.93	0.94	0.96	0.94	0.95	0.97	0.96	0.96	0.97	0.95	0.96
Winter spelt	0.66	0.51	0.58	0.68	0.60	0.64	0.91	0.80	0.85	0.88	0.72	0.79
Winter triticale	0.57	0.36	0.44	0.58	0.45	0.50	0.87	0.72	0.78	0.85	0.71	0.77
Beans	0.91	0.79	0.85	0.95	0.89	0.92	0.95	0.90	0.93	0.94	0.83	0.88
Peas	0.87	0.65	0.75	0.81	0.84	0.82	0.94	0.91	0.92	0.94	0.79	0.86
Potato	0.91	0.92	0.91	0.91	0.92	0.92	0.96	0.96	0.96	0.95	0.94	0.95
Soybeans	0.96	0.84	0.90	0.97	0.82	0.89	0.98	0.90	0.93	0.97	0.87	0.92
Asparagus	0.71	0.92	0.80	0.96	0.85	0.90	0.98	0.94	0.96	0.96	0.97	0.96
Winter wheat	0.94	0.97	0.95	0.93	0.96	0.94	0.96	0.98	0.97	0.96	0.98	0.97
Winter barley	0.94	0.95	0.94	0.93	0.95	0.94	0.96	0.97	0.96	0.96	0.97	0.96
Winter rye	0.84	0.58	0.68	0.82	0.59	0.69	0.94	0.88	0.91	0.94	0.84	0.89
Summer barley	0.85	0.88	0.86	0.85	0.90	0.87	0.92	0.94	0.93	0.94	0.94	0.94
Maize	0.97	0.98	0.97	0.96	0.98	0.97	0.98	0.99	0.98	0.98	0.98	0.98
	OA 93.55%			OA 92.94%			OA 95.99%			OA 95.82%		
	Kappa 0.92			Kappa 0.91			Kappa 0.95			Kappa 0.95		

The classification results on the Talhu Town dataset are presented in **Table 6**. The (3 + 2)D SAFPN model achieved overall accuracies of 89.06% on the validation set and 89.01% on the test set, representing improvements of 2.4% and 2.36%, respectively, over the (3 + 2)D FPN model. The Kappa coefficient also increased from 0.78 to 0.82, indicating enhanced model consistency. Among the crop categories, maize and sunflower exhibited the highest classification performance, with F1-scores exceeding 0.90. In contrast, honeydew melon and sugar beet showed relatively poor performance, with F1-scores ranging between 0.30 and 0.56. According to field investigations, this can be attributed to local agricultural practices where farmers maximize land use by employing inter-row mixed cropping techniques. For example, alternating the planting of sugar beet and custard squash in the same field often leads to spectral confusion, thereby increasing classification difficulty (Qin, 2024). Following the integration of the SA mechanism and Focal Loss, noticeable improvements were observed in the R values of minority classes such as honeydew melon and sugar beet. This demonstrates the proposed method’s enhanced sensitivity and discriminative ability for rare classes.

**Table 6:** Performance comparison of the (3 + 2)D FPN and (3 + 2)D SAFPN models on the test and validation sets of the Talhu Town dataset.

Model	(3 + 2) D FPN						(3 + 2) D SAFPN					
Model_depth	101						50					
Dataset	Talhu town (val set)			Talhu town (test set)			Talhu town (val set)			Talhu town (test set)		
Accuracy Metric	P	R	F1	P	R	F1	P	R	F1	P	R	F1
Maize	0.92	0.93	0.92	0.91	0.93	0.92	0.93	0.95	0.94	0.93	0.95	0.94
Sunflower	0.87	0.95	0.91	0.87	0.95	0.91	0.90	0.95	0.92	0.90	0.96	0.92
Wheat	0.86	0.77	0.81	0.87	0.78	0.82	0.91	0.84	0.88	0.91	0.84	0.87
Honeydew melon	0.70	0.21	0.32	0.67	0.21	0.32	0.75	0.30	0.43	0.73	0.29	0.42
Tomato	0.64	0.49	0.55	0.65	0.47	0.54	0.69	0.54	0.61	0.69	0.53	0.60
Zucchini	0.79	0.76	0.78	0.79	0.77	0.78	0.83	0.82	0.82	0.83	0.81	0.82
Sugar beet	0.60	0.33	0.43	0.61	0.35	0.44	0.69	0.48	0.57	0.69	0.47	0.56
	OA 86.66%			OA 86.65%			OA 89.06%			OA 89.01%		
	Kappa 0.78			Kappa 0.78			Kappa 0.82			Kappa 0.82		

To comprehensively evaluate the classification performance of the models across different crop types, experiments were conducted on both the Munich and Talhu Town datasets using the (3 + 2)D FPN and (3 + 2)D SAFPN models. The classification outcomes were visualized through confusion matrices. The results demonstrate that the (3 + 2)D FPN model performs relatively well for common crop types such as winter wheat, maize, and sunflower, achieving high classification accuracy. However, in scenarios where spectral differences between classes are pronounced or the distribution of training samples is highly imbalanced, the model tends to exhibit confusion, leading to frequent misclassification of rare categories such as sugar beet and custard squash. In contrast, the (3 + 2)D SAFPN model incorporates a SA mechanism and Focal Loss function, which significantly enhance the model’s capability to capture fine-grained features and handle class imbalance. The experimental results indicate that this model exhibits stronger discriminative power in distinguishing spectrally similar crop types. In particular, the R and F1-scores of rare categories are notably improved, thereby boosting overall classification accuracy and robustness.

**Figure 6:**
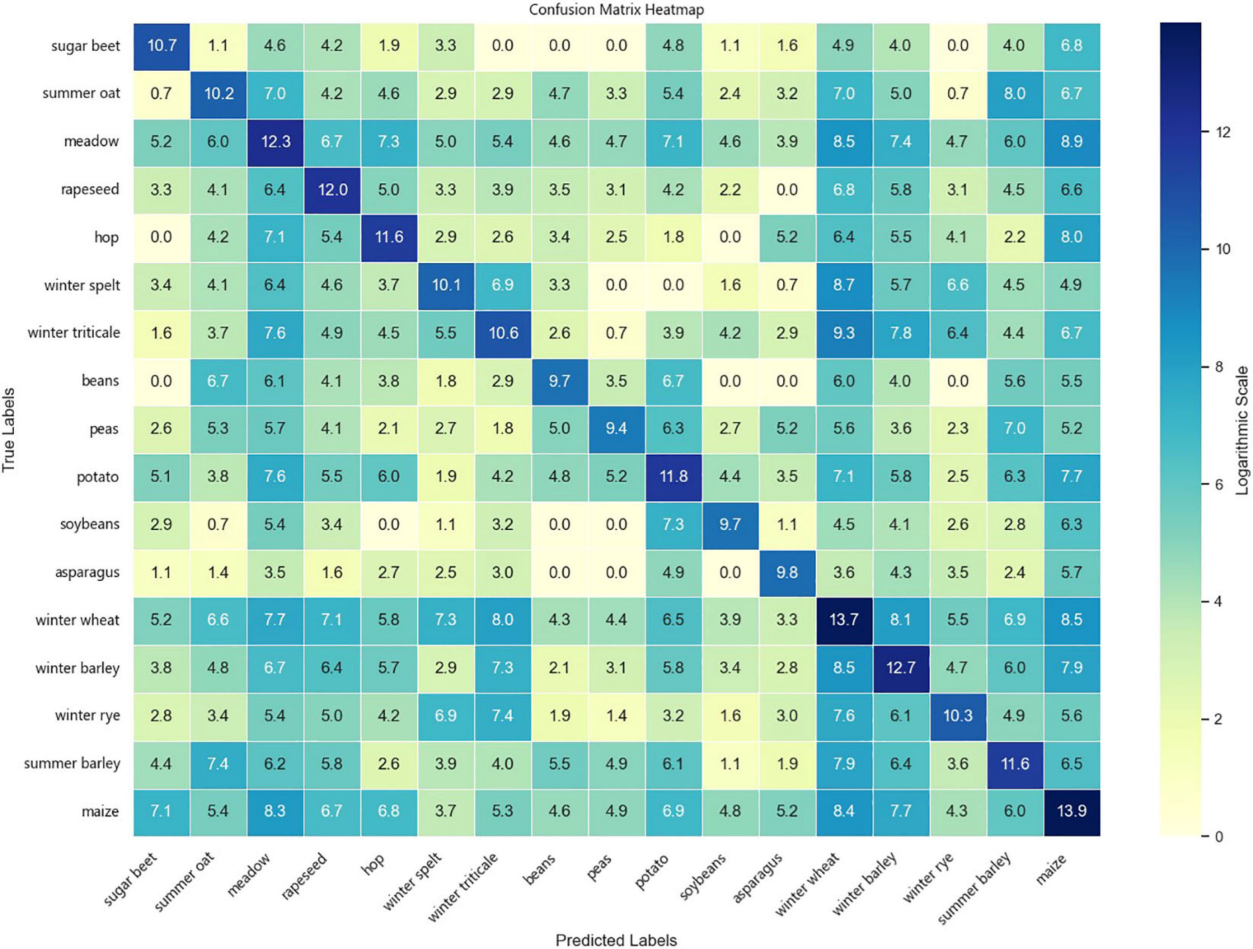
Confusion matrix of the (3 + 2)D SAFPN model on the test set of the Munich dataset.

**Figure 7:**
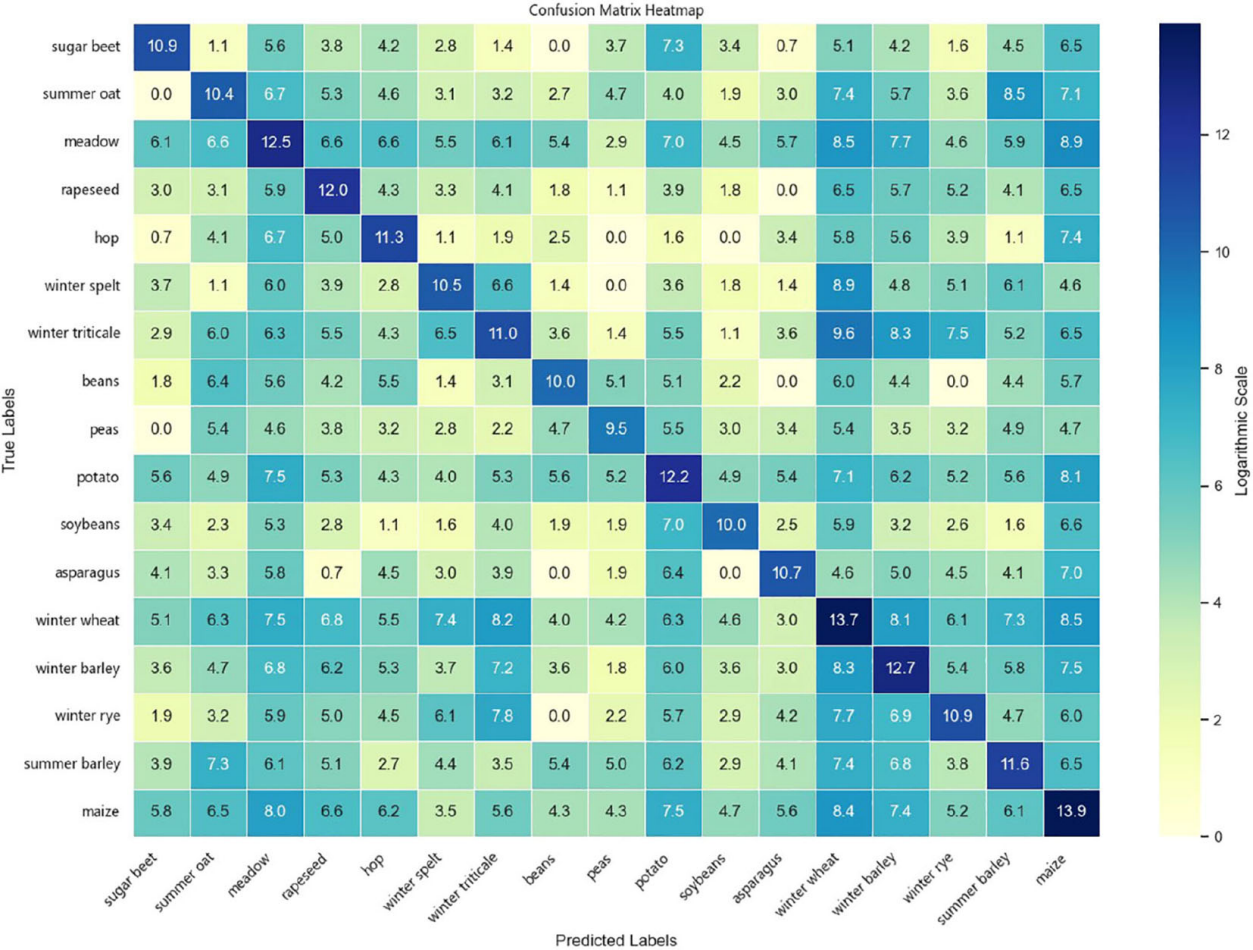
Confusion matrix of the (3 + 2)D SAFPN model on the validation set of the Munich dataset.

**Figure 8:**
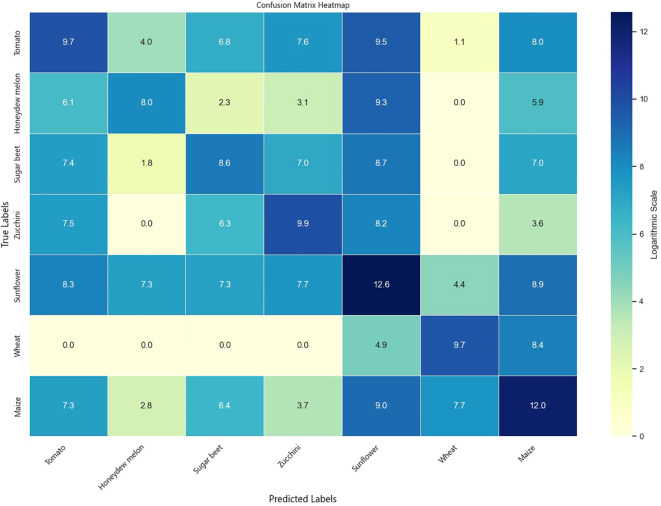
Confusion matrix of the (3 + 2)D FPN model on the test set of the Talhu Town dataset.

**Figure 9:**
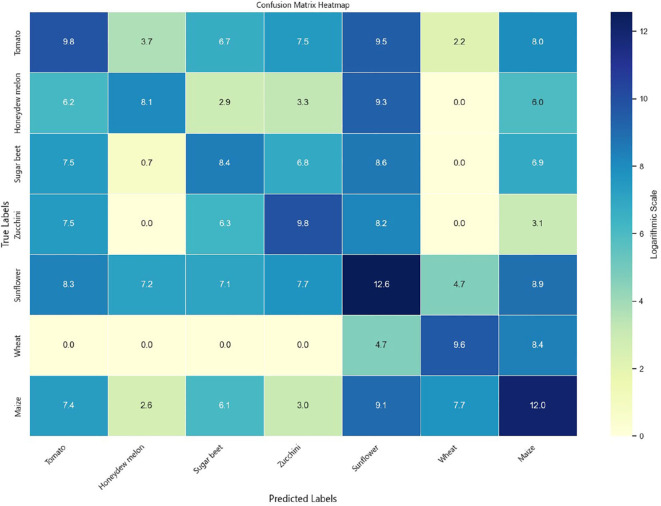
Confusion matrix of the (3 + 2)D FPN model on the validation set of the Talhu Town dataset.

**Figure 10:**
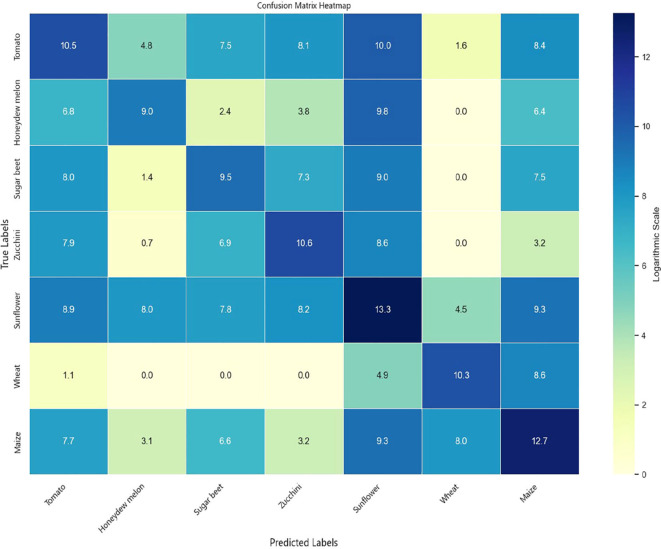
Confusion matrix of the (3 + 2)D SAFPN model on the test set of the Talhu Town dataset.

**Figure 11:**
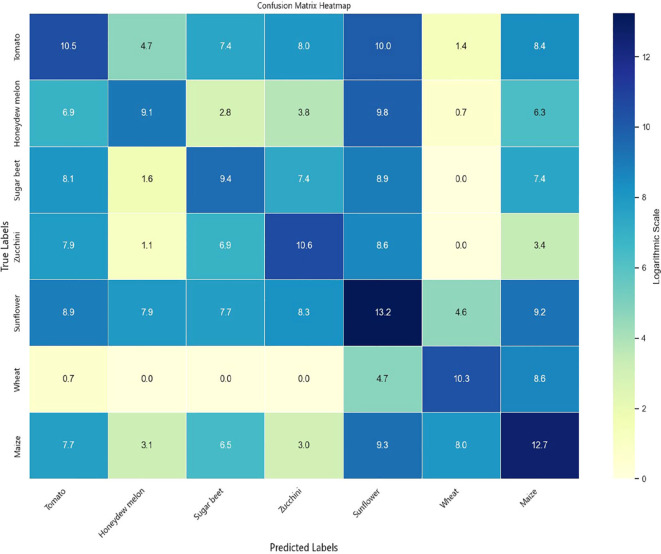
Confusion matrix of the (3 + 2)D SAFPN model on the validation set of the Talhu Town dataset.

**Figure 6** illustrates the confusion matrix of the (3 + 2)D SAFPN model evaluated on the test set of the Munich dataset. **Figure 7** presents the confusion matrix of the (3 + 2)D SAFPN model evaluated on the validation set of the Munich dataset. The confusion matrix of the (3 + 2)D FPN model on the test set of the Talhu Town dataset is shown in **Figure 8**. The confusion matrix of the (3 + 2)D FPN model on the validation set of the Talhu Town dataset is shown in **Figure 9**. The confusion matrix of the (3 + 2)D SAFPN model on the test set of the Talhu Town dataset is shown in **Figure 10**. The confusion matrix of the (3 + 2)D SAFPN model on the validation set of the Talhu Town dataset is shown in **Figure 11**. From these confusion matrices, several observations can be drawn. First, in terms of class-specific discrimination ability, the proposed (3+2)D SAFPN model exhibits clearer diagonal dominance compared with the baseline (3+2)D FPN, indicating improved classification accuracy at the class level. This improvement is particularly evident for spectrally and phenologically similar crop types, such as winter wheat versus winter barley and maize versus summer barley in the Munich dataset. These results suggest that the split-attention mechanism effectively enhances feature selectivity across both temporal and spectral dimensions, enabling the model to better distinguish crops with subtle differences. Second, analysis of the error patterns reveals that misclassifications mainly occur among crop types with overlapping growth cycles and highly similar spectral signatures, as reflected by the off-diagonal elements in the confusion matrices. This phenomenon is consistent with well-known challenges in time-series-based crop mapping and indicates that the remaining classification errors are primarily attributable to intrinsic class similarity rather than model instability or overfitting. Finally, regarding generalization across datasets, a comparison between the Munich and Talhu Town datasets shows that the (3+2)D SAFPN model maintains stable class-wise performance across regions with different cropping structures and agricultural practices. This consistency across datasets demonstrates the robustness of the proposed architecture and supports its strong generalization capability for crop classification tasks under diverse geographic conditions.

### 4.4 Spatial distribution and area statistics of crop planting structure in the study area

A scientifically designed crop rotation system is a critical strategy for achieving sustainable development in modern agriculture. In order to reduce excessive soil nutrient depletion, suppress pests and diseases, improve soil structure, and simultaneously enhance yield and economic benefits, the same crop is not usually cultivated on the same farmland for consecutive years. Instead, an alternate-year rotation scheme is generally adopted (Bennett et al., 2011). Based on this principle, this study validated the spatial distribution of crop classification results for the year 2024 and conducted a comparative analysis with the actual cropping structure derived from the 2022 Jilin-1 satellite imagery.

**Figure 12:**
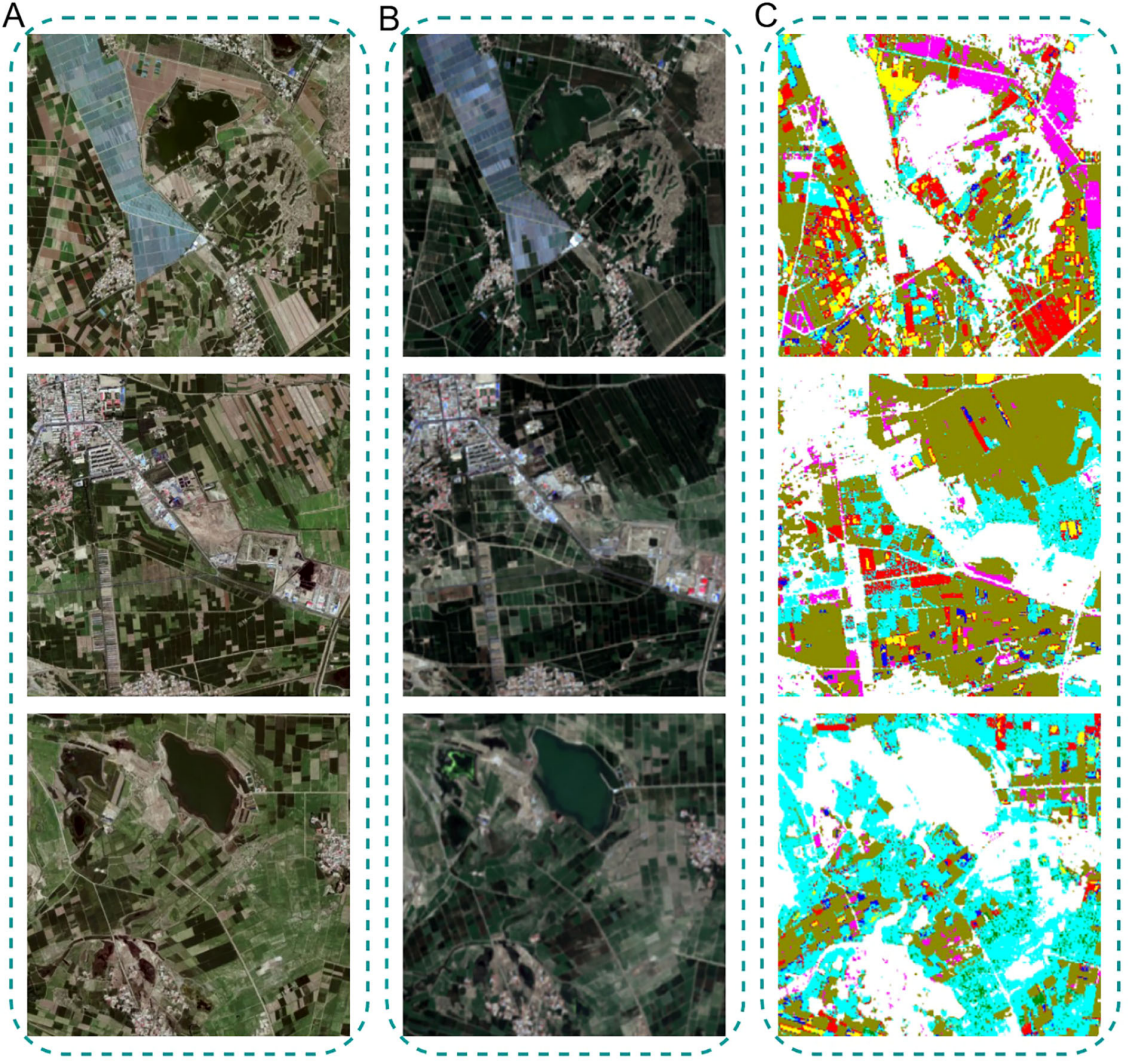
Inference of the main growth stages of crops based on the CAI value.

**Figure 13:**
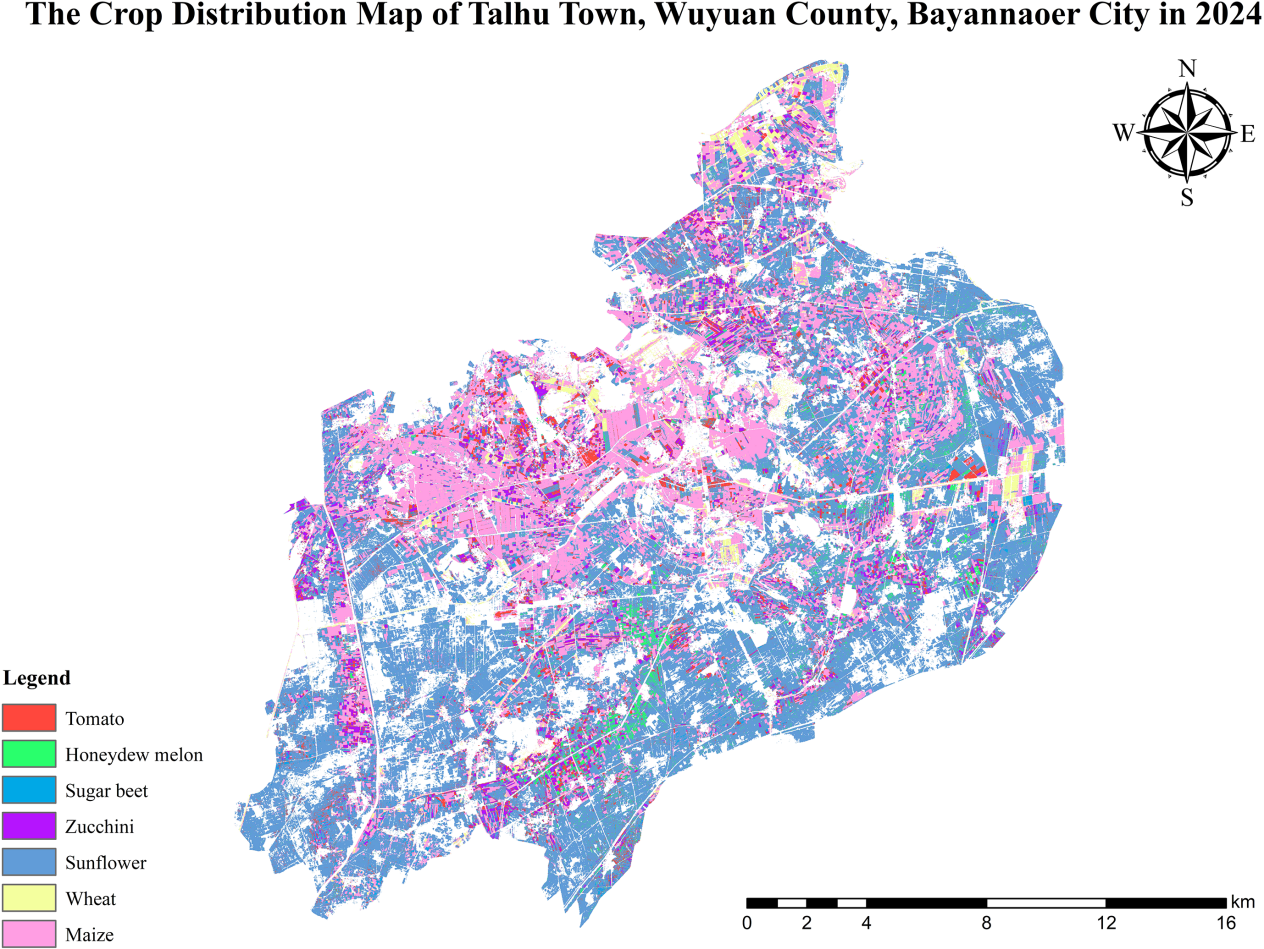
Distribution map of seven crops in Talhu Town in 2024.

**Figure 12** compares the classification results of a selected area in Talhu Town using a 2022 Jilin-1 image (**Figure 12A**) and a 2024 Sentinel-2 image (**Figure 12B**). The classification map (**Figure 12C**) shows that the spatial distribution of the seven labeled crop types and other land cover categories (such as urban areas, sandy land, greenhouses, and water bodies) closely aligns with the actual land use patterns. Within the displayed region, maize and sunflower exhibit a contiguous planting pattern. The classified results reveal that crops such as maize and sunflower are distributed in a parcel-wise manner, which is consistent with the actual cultivated land morphology, further validating the spatial generalization capability of the proposed model. **Figure 13** presents the complete distribution map of seven crop types in Talhu Town for the year 2024, clearly visualizing the spatial distribution of both crops and other land cover types across the study area.

The planting area of each crop can be calculated based on the number of pixels identified for each crop type in the classification results. The area of each pixel is determined by the spatial resolution of the remote sensing imagery, and is equal to the square of the resolution (Li et al., 2023). For instance, if the spatial resolution is 10 meters, each pixel represents an area of 100 m². Taking maize as an example, 890951 pixels correspond to a planting area of 89095100 m², which is approximately 8909.51 hectare. Following this method, the estimated planting areas of the seven major crops in Talhu Town for the year 2024 are listed in **Table 7**.

**Table 7:** Planting areas of seven crops in Talhu Town in 2024.

Crops	Pixel numbers	Planting area(hectare)
Maize	890951	8909.51
Sunflower	1565538	15655.38
Wheat	97623	976.23
Honeydew melon	75673	756.73
Tomato	182398	1823.98
Zucchini	129575	1295.75
Sugar beet	72896	728.96

### 4.5 Parcel-based analysis of NDVI and CAI

**Figure 14:**
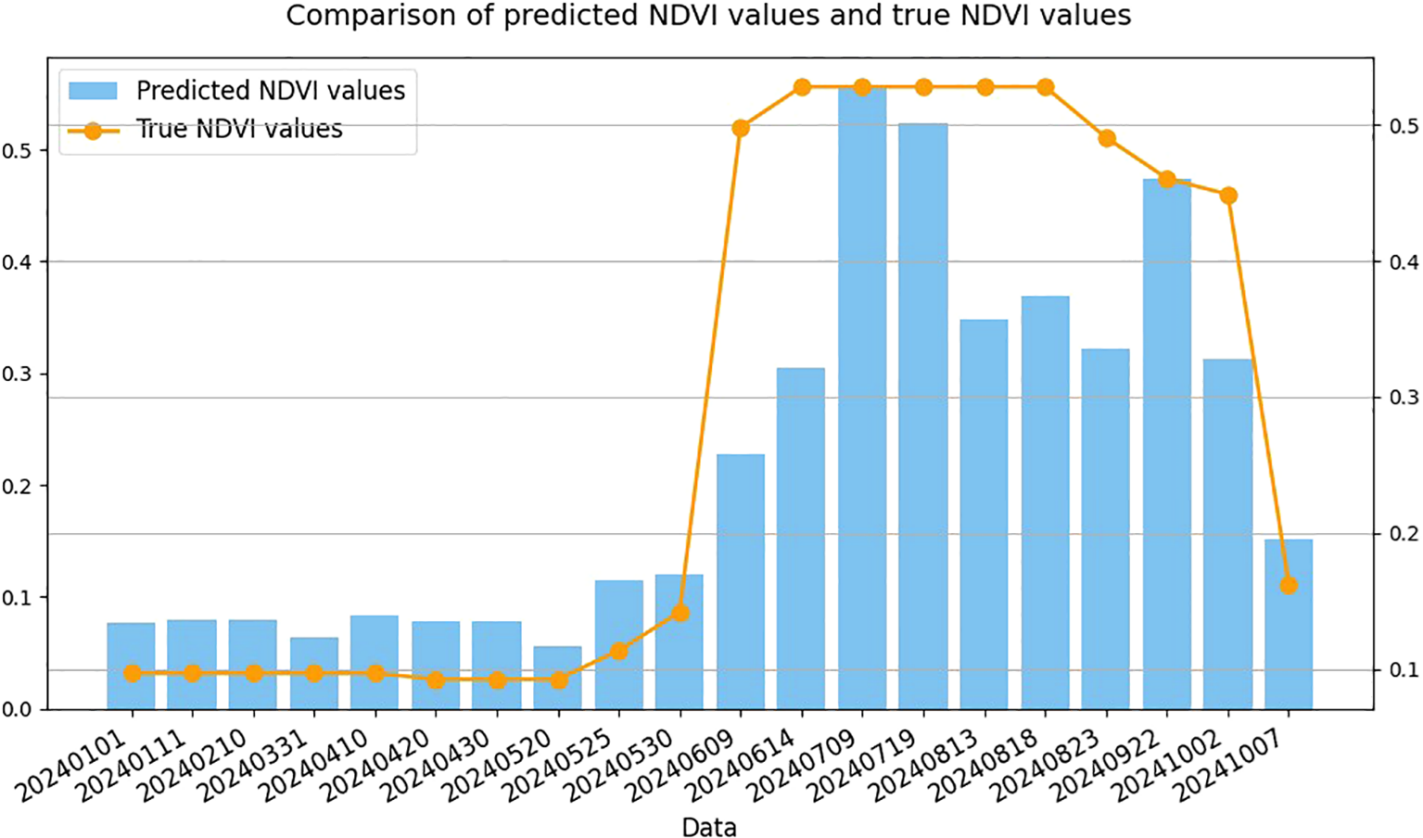
Comparison between the predicted NDVI values by the (3 + 2)D SAFPN model and the real NDVI values.

As an example, a sunflower field randomly selected within the study area was used to evaluate the temporal simulation capability of the (3 + 2)D SAFPN model. **Figure 14** illustrates a comparison between the predicted NDVI values and the observed NDVI values. The predicted NDVI curve is derived from the model’s estimation of temporal sequences during the crop classification process, reflecting its ability to simulate vegetation coverage dynamics over time. In contrast, the observed NDVI values represent the actual growth status of sunflowers at specific time points, as captured by remote sensing. As shown in the figure, the predicted and observed NDVI curves exhibit similar trends across most time periods, particularly during the crop’s critical growth stages, where both curves demonstrate a notable increase. This indicates that the proposed model effectively captures the dynamic growth characteristics of sunflowers and possesses a certain level of temporal simulation capability, which can support subsequent tasks such as crop growth monitoring and dynamic assessment.

**Figure 15:**
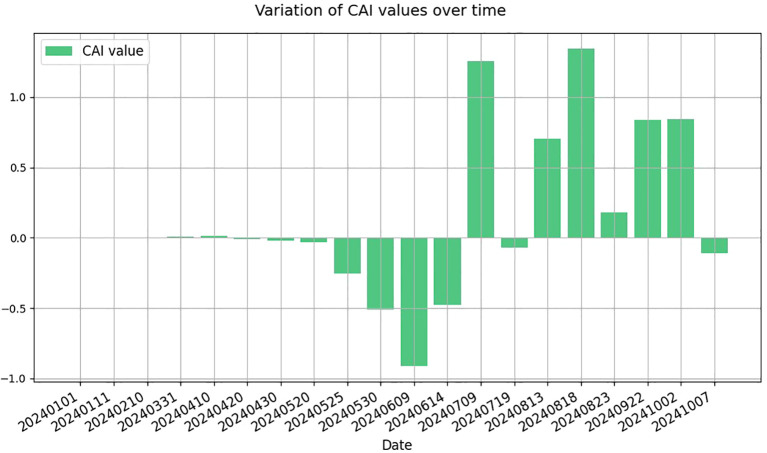
Inference of the main growth stages of crops based on the CAI value.

Furthermore, to reveal the model’s performance in crop recognition across different time phases, **Figure 15** presents the temporal variation of the Category CAI throughout the growth cycle of the selected field. The CAI reflects the model’s activation strength for a specific crop class at each time point, indicating the degree of attention and discriminative ability towards the target category. A positive CAI value suggests a strong model response to the target crop, typically corresponding to vigorous growth stages with distinctive spectral features. Conversely, negative CAI values indicate weaker activation, possibly due to early sowing stages, indistinct growth characteristics, or interference from weeds and mixed crops.

**Figure 16:**
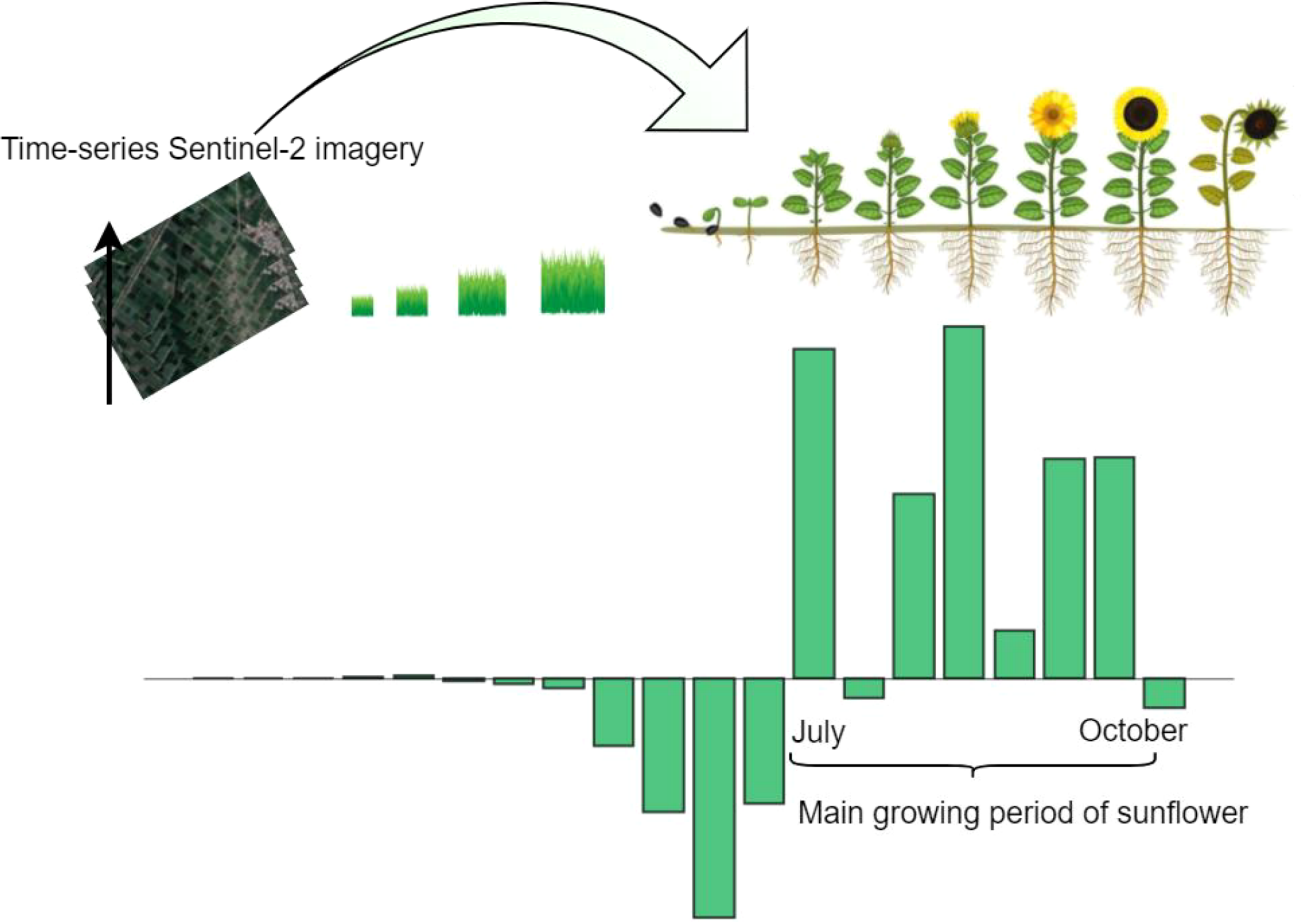
Inference of the main growth stages of crops based on the CAI value.

As shown in **Figure 16**, from early July to early October, CAI values remain in the positive range, suggesting that the model effectively captures key features and maintains stable recognition performance during the sunflower’s main growth period. In contrast, at the early sowing or seedling stages, CAI values are generally low or negative due to limited vegetation cover and weak spectral differentiation, revealing the model’s limitations in early-stage recognition. As the crop enters the rapid growth phase, spectral features become more prominent, leading to a rise in CAI values that peak at the maturity stage. This trend aligns closely with the actual phenological development of sunflowers, demonstrating the model’s effectiveness and reliability in time-series crop classification.

## 5 Discussion

Multi-temporal remote sensing data of crops present significant challenges for large-scale crop classification due to their high heterogeneity in both spectral and spatial dimensions. Although crop growth exhibits strong temporal correlations, many existing models fail to fully exploit this characteristic, often adopting simplified representations of temporal information. For instance, traditional methods such as SVM and RF treat each time step in the sequence as an independent dimension, typically overlooking the correlations within the sequence and relying on overly simplified heuristic rules to handle temporal features (Kamilaris and Prenafeta-Boldú, 2018). Consequently, these models struggle to effectively utilize temporal information. 2D CNNs perform convolutions only along the spatial dimension. While they are effective at extracting spatially relevant features, their limited capacity to model temporal dynamics leads to suboptimal classification accuracy—especially when distinguishing crops with similar spectral signatures but distinct phenological patterns. Although SVM and RF methods can leverage spectral information, they are inherently incapable of capturing complex spatiotemporal variations (Verhulst et al., 2024). In contrast, convolutional neural networks based on image patches can extract spatial features and deep-level representations (Wang and Zuo, 2024). However, conventional 2D CNNs still struggle to capture the temporal variability in multi-temporal and multi-spectral datasets, limiting their classification performance (Cheng et al., 2023). Unlike 2D CNNs, 3D CNNs utilize 3D convolutional kernels that operate jointly over spatial and temporal dimensions, enabling them to better extract spatiotemporal features from multi-temporal remote sensing data. As a result, 3D CNNs outperform traditional models such as 2D CNNs, SVMs, and RF in classification accuracy (Ma et al., 2023). Nevertheless, relying solely on either 3D CNNs or 2D CNNs remains insufficient for fully integrating spatial and temporal information.

To address the aforementioned challenges, this study proposes a (3 + 2)D SAFPN model designed to jointly capture the spatiotemporal dynamics and multi-scale spatial features of crop growth. The 3D FPN is employed to model the spatiotemporal characteristics of multi-temporal remote sensing data, while the 2D FPN enhances spatial feature representation across different scales through a feature pyramid structure. The combination of the two enables the model to effectively leverage both temporal variation during crop development and spatial-scale information. Additionally, a SA mechanism is incorporated to dynamically reweight feature channels, thereby improving the model’s feature selection capability. Originating from the ResNeSt architecture, the SA mechanism divides feature maps into multiple subgroups and applies channel-wise weighting to each subgroup, enhancing the model’s ability to express key features. Integrating this mechanism into both the 3D and 2D FPN structures allows the model to better handle multi-scale features and perform more precise feature selection in complex spatiotemporal data. To address the class imbalance inherent in remote sensing imagery, this study adopts Focal Loss in place of conventional CE Loss. While CE Loss assigns equal weight to all samples, it often leads to a bias toward dominant classes. In contrast, Focal Loss reduces the influence of easily classified samples and emphasizes harder examples, thereby improving the model’s sensitivity and discriminative ability for minority classes and achieving more balanced classification results. With these enhancements, the proposed (3 + 2)D SAFPN model demonstrates improved performance in spatiotemporal feature extraction, multi-scale representation, and class imbalance mitigation. In particular, when dealing with multi-temporal remote sensing data, the model more accurately captures feature information across different stages of crop growth. Compared to standalone 2D CNN, SVM, or RF models, the proposed architecture achieves superior performance and offers a more efficient solution for crop classification tasks.

Experimental results on the Munich dataset demonstrate that, compared to the traditional (3 + 2)D FPN model, the proposed method improves classification accuracy on the test and validation sets by 2.88% and 2.44%, respectively. Classification accuracy for dominant crops such as sugar beet, maize, and winter barley exceeds 90%. For crops with similar spectral properties and growth cycles, such as peas and winter rye, the SA mechanism enhances the interaction between feature channels, significantly improving the model’s ability to distinguish in fine-grained classification tasks. Experiments on the Talhu Town time-series NDVI dataset confirm the model’s generalization ability, with the (3 + 2)D SAFPN model increasing classification accuracy on the test and validation sets by 2.36% and 2.4%, respectively. Among the crops with better classification performance, maize, sunflower, wheat, and zucchini achieved classification accuracies above 81%. However, crops like honeydew melon and sugar beet, which are often grown in double-row or mixed planting patterns, exhibit spectral confusion with neighboring crops, resulting in difficulties in recognition. To address this, the introduction of the SA mechanism and Focal Loss effectively enhances the model’s feature selection capability and optimizes the handling of class imbalance, improving recognition performance for crops like sugar beet and honeydew melon.

To further validate the model’s ability to predict crop spatial distribution, the classification results for seven crops in Talhu Town from the 2024 Sentinel-2 imagery were compared with the actual planting structure derived from the 2022 Jilin-1 satellite imagery. The results indicate that the spatial distribution of major crops such as maize and sunflower aligns closely with the real planting patterns, further confirming the model’s reliability in spatial aspects. By comparing the NDVI time series generated from the classification results with actual NDVI values, it was found that the model exhibits strong fitting ability for the NDVI variation trends at key growth stages, reflecting its capability in modeling crop growth dynamics. However, NDVI has certain inherent limitations, such as sensitivity to soil background, saturation under high vegetation cover, and limited capability to fully discriminate spectrally similar crop types. Future research could consider integrating additional spectral indices, such as the Enhanced Vegetation Index (EVI), Soil-Adjusted Vegetation Index (SAVI), or Normalized Difference Water Index (NDWI), to provide richer spectral information and further improve crop classification accuracy as well as the identification of complex cropping patterns. Additionally, analysis of the temporal variation of CAI values shows that the model demonstrates high timeliness and accuracy in recognizing crop classes during the main growth stages.

In summary, the proposed (3 + 2)D SAFPN model demonstrates good performance on both the Munich and Talhu Town datasets. By integrating 3D and 2D feature pyramid structures, incorporating the SA mechanism, and utilizing Focal Loss, the model enhances the expression of multi-scale features and spatiotemporal information, effectively alleviating the class imbalance problem and improving the classification accuracy of complex crops, especially in cases of mixed planting and complex temporal features. Future research will continue to explore the model’s generalization ability on larger regions, multiple crop types, and multi-modal remote sensing data, and will attempt to integrate self-supervised learning strategies to further reduce dependence on labeled data, providing more practical technological support for agricultural remote sensing monitoring and intelligent decision-making.

## 6 Conclusion

Fully leveraging the phenological variations embedded in multi-temporal remote sensing data to improve crop classification and mapping accuracy is a key research direction in agricultural remote sensing. This study focuses on the main crop types in Talhu Town, Wuyuan County, Bayannur City, Inner Mongolia Autonomous Region. Based on Sentinel-2 time-series imagery, a parcel-level NDVI dataset was constructed, and a novel (3 + 2)D SAFPN model was proposed for fine-grained crop classification and planting structure extraction. The model achieved precise recognition of various crops at the parcel scale. Experimental results demonstrated that it outperforms the traditional (3 + 2)D FPN model across different datasets, showcasing strong generalization and application potential. The main conclusions of this study are as follows: 1) Construction of a parcel-level NDVI time-series dataset for Talhu Town: This study selected optimal Sentinel-2 images at representative time points in 2024 to build an NDVI time-series dataset covering the entire crop growth season. This approach effectively captures the phenological dynamics of crop growth. The dataset integrates multi-temporal and multi-resolution remote sensing information, enabling accurate differentiation of crop growth stages and significantly enhancing the temporal quality of input features for classification models. Moreover, this method is not only applicable to the Talhu Town area but also demonstrates strong transferability and generalizability, providing a reliable data foundation for agricultural monitoring and ecological assessment in other regions. 2) Proposal of the (3 + 2)D SAFPN model: The proposed (3 + 2)D SAFPN model integrates 3D and 2D feature pyramid networks to fully extract spatiotemporal and multi-scale features of crops. A SA mechanism is introduced to dynamically reweight feature channels according to their importance, enhancing feature representation. Meanwhile, the incorporation of the Focal Loss function reduces the influence of easily classified samples, improving the model’s ability to recognize minority crop classes and effectively mitigating class imbalance. Experiments show that this model not only improves classification accuracy but also optimizes memory usage efficiency, making it suitable for large-scale remote sensing applications. 3) Validation of model adaptability and robustness across datasets: On the Munich public dataset, the (3 + 2)D SAFPN model achieved accuracy improvements of 2.88% and 2.44% on the test and validation sets, respectively, and performed particularly well in distinguishing confusing crops such as winter wheat and winter rye. On the Talhu Town dataset, classification accuracy improved by 2.36% and 2.40% on the test and validation sets, respectively. The model achieved high accuracy for major crops such as maize and sunflower. Although crops like melon and tomato pose classification challenges due to inter-row or mixed planting patterns, the integration of the Split-Attention mechanism and Focal Loss significantly enhanced recognition performance for sugar beet and melon, further validating the model’s effectiveness and robustness under complex planting structures.

## Data Availability

The datasets presented in this study can be found in online repositories. The names of the repository/repositories and accession number(s) can be found below: https://gitee.com/btgw/YicongSun/ree/(3+2)D-SAFPN_torch.
